# New single-nucleotide polymorphisms associated with differences in platelet reactivity and their influence on survival in patients with type 2 diabetes treated with acetylsalicylic acid: an observational study

**DOI:** 10.1007/s00592-016-0945-y

**Published:** 2016-12-19

**Authors:** Lukasz Milanowski, Justyna Pordzik, Piotr K. Janicki, Agnieszka Kaplon-Cieslicka, Marek Rosiak, Michal Peller, Agata Tyminska, Krzysztof Ozieranski, Krzysztof J. Filipiak, Grzegorz Opolski, Dagmara Mirowska-Guzel, Marek Postula

**Affiliations:** 1 Department of Experimental and Clinical Pharmacology, Center for Preclinical Research and Technology CEPT, Medical University of Warsaw, Banacha 1B str., 02-097 Warsaw, Poland; 2 Perioperative Genomics Laboratory, College of Medicine, Penn State University, Hershey, PA USA; 3 Department of Cardiology and Hypertension, Central Clinical Hospital, Ministry of the Interior, Warsaw, Poland; 4 Department of Cardiology, Medical University of Warsaw, Warsaw, Poland

**Keywords:** Diabetes, Genetics, Acetylsalicylic acid, Platelet reactivity

## Abstract

**Aims:**

Genetic polymorphisms may contribute to platelet reactivity in diabetic patients; however, the information on their influence on long-term antiplatelet therapy is lacking. Our aim was to evaluate the role of previously described genetic variants and platelet reactivity on risk of all-cause mortality and cardiovascular events.

**Methods:**

Blood samples were obtained from 303 Caucasian patients. Genome-wide genotyping was performed using Illumina Human Omni 2.5-Quad microarrays, and individual genotyping of selected SNPs was performed using a custom Sequenom iPLEX assay in conjunction with the Mass ARRAY platform. Platelet reactivity was measured with VerifyNow Aspirin Assay and PFA-100 Assay. Univariate and multivariate Cox regression analyses were performed to determine the impact of genetic variants and platelets reactivity on risk of all-cause mortality and cardiovascular events.

**Results:**

Among the 237 patients included in the follow-up, death from any cause occurred in 34 (14.3%) patients and cardiovascular events occurred in 51 (21.5%) patients within a median observation time of 71 months (5.9 years). In univariate analyses, significant association in the presence of minor alleles in TXBA2R (rs1131882) with primary (HR 2.54, 95% CI 1.15–5.60, *p* = 0.021) and secondary endpoint (HR 2.06, 95% CI 1.06–4.04, *p* = 0.034) was observed. In addition, multivariate analyses revealed the impact of this polymorphism on primary (HR 2.34, 95% CI 1.09–5.00, *p* = 0.029) and secondary endpoint (HR 1.89, 95% CI 1.00–3.57, *p* = 0.048).

**Conclusions:**

Results of the study demonstrate for the first time an association between genetic polymorphism within TXBA2R gene encoding platelet’s surface receptor and long-term survival of diabetic patients treated with ASA.

## Introduction

The number of people with diabetes, a chronic and lifelong condition, is dangerously high due to the growing prevalence of obesity, genetic susceptibility, urbanization and aging [1]. The prevalence of this disease has soared in Europe over the past decade, increasing by more than 50% in many countries (European Cardiovascular Disease Statistics, 2012 edition). Type 2 diabetes mellitus (T2DM), which is the most common form of diabetes, is often diagnosed incidentally at an advanced stage when cardiovascular events have already occurred in most patients [1].

Platelets are equipped with multiple receptors which take part in activating the physiological response of platelets through specific agonists and if activated, they show an increased adhesiveness and aggregation [2]. The hypercoagulable state in T2DM might be a consequence of disturbances in the processes of platelet activation, aggregation and lifespan, as well as the pro-inflammatory state and endothelial dysfunction [3, 4].

Recent advances in antiplatelet therapy for cardiovascular risk reduction do not seem to affect patients with T2DM, who continue to experience a higher risk of ischemic events than non-diabetic patients treated with acetylsalicylic acid (ASA) [5]. Type 2 diabetes mellitus was also found to be associated with higher platelet reactivity in spite of ASA therapy [6–9]. Grimaldi et al. [10] on the other hand reported that a significant number of diabetic patients have increased platelet reactivity despite ASA treatment, which is further associated with poor prognosis.

The genetic background of platelet activation may be of great importance in establishing the cause of inter-individual differences in platelet reactivity in T2DM population treated with ASA. Several single-nucleotide polymorphisms (SNPs) could be responsible for at least some of these differences. Results from our previous study suggested that 4 genomic loci [TXBA2R (rs1131882), ADRA2A (rs4311994), PLA2G7 (rs7756935) and 9p21.3 (rs10120688)] may contribute to platelet reactivity in this population [2]. In another study, in which genome-wide association approach (GWAS) and pooled DNA samples strategy were used, the most significant results were obtained for rs2502448 located in the intronic part of RGS7 gene on chromosome 1. Two other SNPs were found to be significant, DPP6 (rs1387180) and GRS (rs3779647), and thus may be potentially associated with inter-individual differences in platelet activation measured in the cohort of diabetic patients on ASA treatment [9].

As the information on impact of genetic polymorphisms associated with platelet reactivity in stable T2DM patients on long-term antiplatelet therapy is lacking, we decided to evaluate the role of the previously described SNPs on the risk of all-cause mortality and cardiovascular events in this high-risk population. We also aimed to investigate the role of platelet reactivity on occurrence of these events by measuring PFA-100 collagen/epinephrine closure time (CEPI-CT) and/or collagen/ADP closure time (CADP-CT) and/or VerifyNow aspirin reaction units (ARU).

## Methods

The ethics committee of the Medical University of Warsaw approved both the study protocol and the informed consent form. The study was conducted in accordance with the current version of the Declaration of Helsinki at the time when the study was designed, and informed written consent was obtained. The genotyping part of the study was reviewed and approved by the Institutional Review Board of Penn State Hershey Medical Center (Hershey, PA, USA). The study subjects were recruited consecutively from patients with T2DM [participating in a multi-center, prospective, randomized and open-label AVOCADO (Aspirin vs./Or Clopidogrel in Aspirin resistant Diabetics inflammation Outcomes) study] presenting to the outpatient clinic of the Central Teaching Hospital of the Medical University of Warsaw. The full characterization of the study population, including the inclusion and exclusion criteria, was published previously [2]. Briefly, the Caucasian subjects with T2DM who, at the time of enrollment, had been taking ASA tablets at the dose of 75 mg per day for at least 3 months for primary or secondary prevention of myocardial infarction (MI) were recruited. Neither clopidogrel nor antiplatelet drugs other than ASA were used in any of the investigated patients. All patients had been taking oral antidiabetic agents and/or insulin for at least 6 months; diet-controlled diabetic patients were not included. Compliance to ASA therapy at the study entry was based upon the patient’s own statement and serum thromboxane B2 (S-TXB2) level measurement.

The primary endpoint was defined as death from any cause. The secondary endpoint included primary endpoint and thromboischemic events, such as ischemic stroke, myocardial infarction, transient ischemic attack, acute ophthalmic artery thrombosis and acute lower limb thrombosis. Information on vital status was obtained from the Central Statistical Office of Poland and hospital records. The interview (previously approved by our medical center protocol) about hospitalization due to thromboischemic events or any other reason during the follow-up period was conducted by direct phone contact with all participants that agreed to participate in follow-up.

Blood samples were taken in the morning 2–3 h after the last ASA dose. Detailed information on blood sample and assay procedures was previously described [2, 8, 9].

Platelet reactivity was measured with VerifyNow Aspirin Assay (Accumetrics, San Diego, CA, USA) and PFA-100 assay (Dade-Behring International, Inc., Newark, DE, USA). All measurements were taken only once, at the beginning of the observational period. These assays were performed in detail as described earlier. Based on our own and other previous reports, we used 3 cutoff values for high platelet reactivity: in the CEPI-CT assay <193 s, in the collagen/adenosine diphosphate CADP-CT < 90 s and in the ARU ≥ 550 [2, 11–13].

Genetic analysis was performed as previously described [2, 9]. DNA, obtained from whole blood samples, was stored in frozen form until analysis which employed the membrane ultrafiltration method using a FujiMiniGene 80 extractor (FujiFilm Life Sciences distributed by Autogene, Holliston, MA, USA). Genome-wide genotyping was performed using Illumina Human Omni 2.5-Quad microarrays (Illumina Inc., San Diego, CA, USA) using Infinium LCG protocol and according to the manufacturer’s recommendations. Microarrays were scanned using IlluminaHiScan system by Beckman Coulter Genomics (Morrisville, NC, USA) and raw data were extracted for statistical analysis with Illumina GenomeStudio v.2010.3 software (Illumina Inc., San Diego, CA, USA). Individual genotyping of selected SNPs (both from GWAS experiment and previously selected from the literature) was performed at the Children’s Hospital Boston using a custom Sequenom iPLEX assay in conjunction with the Mass ARRAY platform (Sequenom Inc., La Jolla, CA, USA).

Categorical data were presented as number of patients and percentages. Normally distributed continuous variables were presented as mean value and standard deviation. For ordinal variables and non-normally distributed continuous variables, median value and interquartile range (IQR) were used. To determine differences between groups, Fisher’s exact test was performed for categorical variables and Mann–Whitney *U* test for continuous and ordinal variables. Kaplan–Meier curves were developed for the primary and secondary endpoint in the two groups. Univariate and multivariate analyses for 5-year survival were performed with a Cox proportional hazards model, which was selected in a backward stepwise manner. Due to a relatively small size of the study groups, and to maintain adequate events per predictor variable (EPV) value, we included only the most important clinical data into the analysis (age, sex, dyslipidemia, history of previous stroke, MI, hypertension, CHF, CAD).

Statistical significance was considered for *p* values lower than 0.05 for all tests. All tests were two tailed. Statistical analyses were performed using Statistica, version 12 (StatSoft, Inc.).

The statistical power calculations were executed by assistance of the online power calculator available at https://www.dssresearch.com/KnowledgeCenter/toolkitcalculators/statisticalpowercalculators.aspx. The null hypothesis for the study has been that there is no statistically significant difference (*p* < 0.05, based on two-tailed Fisher’s exact test) in MAF for single investigated SNP between the survivors and non-survivors in the study cohort at the end of the observation period. Based on the available data, we determined that in the cohort of available 237 patients and observed 14.3% mortality rate, there is 79% power to detect twofold difference in MAF for the investigated SNP between survivors and non-survivors, with observed MAF for investigated SNP of 0.25, and hence we reject the null hypothesis.

## Results

Clinical and demographic characteristics, as well as platelet function tests, are presented in Table 1 (detailed characteristics of population included into the AVOCADO study was previously described [2, 9]), whereas genotyping results are presented in Table 2.

**Table 1:** Clinical and demographic characteristics with platelet function analysis of patients included in AVOCADO study (*n* = 303)

Clinical characteristic	Number of patients
Sex (females)	139 (47.1%)
Age	67.3 ± 8.8
Hypertension	273 (92.5%)
CAD	165 (55.9%)
Dyslipidemia	242 (82.0%)
CHF	111 (37.6%)
MI	89 (30.2%)
Ischemic stroke	24 (8.1%)
CEPI-CT (s)	474.43 ± 71.3
CADP-CT (s)	232.26 ± 73.7
VerifyNow (ARU)	126.07 ± 76.03

*CAD* coronary artery disease, *CHF* congestive heart failure, *MI* myocardial infarction, *CEPI*-*CT* collagen/epinephrine closure time by PFA-100 method, *CADP*-*CT* collagen/adenosine diphosphate closure time by PFA-100 method, *ARU* aspirin reaction units for VerifyNow

**Table 2:** Allele distribution and number of genotyped patients

Gene	Allele frequency	Number of subjects
GRS (rs3779647)	CC/CT/TT 0.2/0.48/0.32	CC/CT/TT 47/111/74
DPP6 (rs1387180)	GG/AG/AA 0.08/0.34/0.58	GG/AG/AA 19/79/135
RGS7 (rs2502448)	CC/CT/TT 0.17/0.5/0.33	CC/CT/TT 39/116/76
PLA2G7 (rs7756935)	CC/AC/AA 0.01/0.29/0.7	CC/AC/AA 2/64/154
Chr 9p21.3 (rs10120688)	AA/AG/GG 0.19/0.51/0.30	AA/AG/GG 41/109/64
TXBA2R (rs1131882)	AA/AG/GG 0.02/0.23/0.75	AA/AG/GG 4/51/165
ADRA2A (rs4311994)	TT/CT/CC 0.02/0.15/0.83	TT/CT/CC 4/33/180

*GRS* glutathione reductase, *DPP6* dipeptidyl peptidase like 6, *RGS7* regulator of G-protein signaling 7, *PLA2G7* lipoprotein-associated phospholipase A2 (Lp-PLA2)/plasma platelet-activating factor acetylhydrolase (PAF-AH), *Chr 9p21.3* chromosome 9p21.3, *TXBA2R* thromboxane A2 receptor, *ADRA2A* alpha-2A-adrenergic receptor

Out of 303 patients initially enrolled to AVOCADO study, 66 subjects were unwilling to participate or were lost in follow-up. Among the 237 patients included in the follow-up, primary endpoint occurred in 34 (14.3%) patients and secondary endpoint occurred in 51 (21,5%) patients within a median observation time of 71 months (5.9 years) (Table 3). At least one event was necessary to classify a patient into the secondary endpoint group. Out of all subjects included in the follow-up, ischemic stroke occurred in 5 (2.1%), myocardial infarction in 9 (3.8%), transient ischemic attack in 1 (0.4%), acute ophthalmic artery thrombosis in 1 (0.4%) and acute lower limb’s artery occlusion in 1 patient (0.4%).

**Table 3:** Baseline comparison of the study group

	Primary endpoint (+) *N* = 34	Primary endpoint (−) *N* = 203	*p* value	Secondary endpoint (+) *N* = 51	Secondary endpoint (−) *N* = 186	*p* value
Sex (females)	11 (32.4%)	95 (47.0%)	0.14	22 (43.1%)	84 (45.4%)	0.87
Age	73 (66.75–77)	67 (60–73.5)	0.001	72 (64.5–76)	67.5 (60–74)	0.018
Hypertension	32 (94.1%)	181 (92.3%)	1.00	49 (96.1%)	164 (91.6%)	0.37
CAD	23 (67.6%)	107 (54.6%)	0.19	35 (68.6%)	95 (53.1%)	0.06
Dyslipidemia	26 (76.5%)	161 (82.1%)	0.48	42 (82.4%)	145 (81.0%)	1.00
CHF	19 (55.9%)	69 (35.2%)	0.03	25 (49.0%)	63 (35.2%)	0.1
MI	16 (47.1%)	58 (29.6%)	0.05	23 (45.1%)	51 (28.5%)	0.03
Ischemic stroke	5 (14.7%)	16 (8.2%)	0.21	6 (11.8%)	15 (8.4%)	0.42
CEPI-CT^a^	9 (26.5%)	50 (25.5%)	1.00	16 (31.4%)	43 (24.0%)	0.28
CADP-CT^a^	13 (38.2%)	82 (41.8%)	0.85	18 (35.3%)	77 (43.0%)	0.34
ARU^a^	11 (32.4%)	44 (22.4%)	0.27	16 (31.4%)	39 (21.8%)	0.19
GRS (rs3779647)^b^	26 (78.8%)	132 (66.7%)	0.22	35 (71.4%)	123 (67.6%)	0.73
DPP6 (rs1387180)^b^	16 (47.1%)	82 (41.8%)	0.58	19 (38.0%)	79 (43.9%)	0.52
RGS 7 (rs2502488)^b^	28 (84.8%)	127 (64.5%)	0.03	38 (77.6%)	117 (64.6%)	0.12
PLA2G7 (rs7756935)^b^	10 (29.4%)	56 (30.3%)	1.00	14 (27.5%)	52 (31.0%)	0.73
Chr 9p21.3 (rs10120688)^b^	23 (67.6%)	127 (70.9%)	0.69	32 (66.7%)	118 (71.5%)	0.59
TXBA2R (rs1131882)^b^	12 (36.4%)	43 (23.4%)	0.13	16 (32.0%)	39 (23.4%)	0.27
ADRA2A (rs4311994)^b^	2 (6.3%)	35 (18.9%)	0.12	7 (14.3%)	30 (17.9%)	0.67

*CAD* coronary artery disease, *CHF* congestive heart failure, *MI* myocardial infarction, *CEPI*-*CT* collagen/epinephrine closure time by PFA-100 method, *CADP*-*CT* collagen/adenosine diphosphate closure time by PFA-100 method, *ARU* aspirin reaction units for VerifyNow, *GRS* glutathione reductase, *DPP6* dipeptidyl peptidase like 6, *RGS7* regulator of G-protein signaling 7, *PLA2G7* lipoprotein-associated phospholipase A2 (Lp-PLA2)/plasma platelet-activating factor acetylhydrolase (PAF-AH), *Chr 9p21.3* chromosome 9p21.3, *TXBA2R* thromboxane A2 receptor, *ADRA2A* alpha-2A-adrenergic receptor

^a^Measured for different closure time cutoff points (CEPI-CT < 193 s, CADP-CT < 90 s, VerifyNow ARU ≥ 550)

^b^Carriers of one or more minor allele (dominant model)

**Fig. 1:**
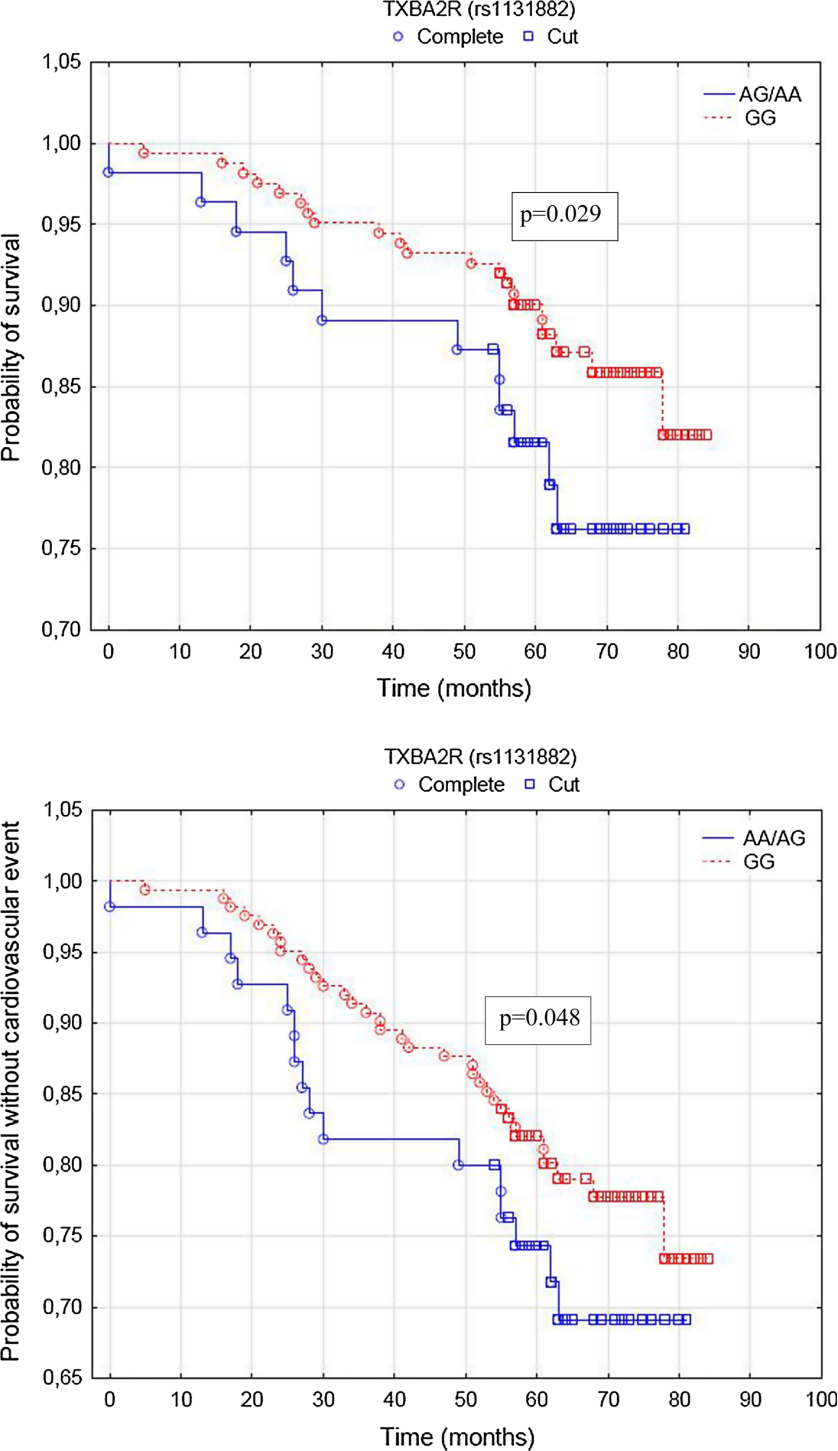
Influence of TXBA2R (rs1131882) minor allele carriers on long-term survival and survival without cardiovascular event

In a univariate analysis, significant association was found in the presence of minor alleles (dominant model) in TXBA2R (rs1131882) and age (Table 4). In univariate analysis, no association was established between platelet reactivity measured with CADP-CT (*p* = 0.928), CEPI-CT (*p* = 0.606) and ARU (*p* = 0.125) and primary endpoint (Table 4). In multivariate regression model, the rs1131882 polymorphism (*p* = 0.029) and age (*p* = 0.0009) correlate with mortality (Table 5). Kaplan–Meier surviving curves for the dominant model of rs1131882 polymorphism are shown in Fig. 1.

**Table 4:** Univariate analysis for primary and secondary endpoint

Variables	Primary endpoint		Secondary endpoint	
	HR (95% Cl)	*p* value	HR (95% Cl)	*p* value
Age	1.074 (1.024–1.125)	**0.003**	1.045 (1.007–1.084)	**0.019**
Sex (females)	0.452 (0.203–1.007)	0.052	0.936 (0.504–1.741)	0.835
Hypertension	1.493 (0.292–7.651)	0.630	2.114 (0.461–9.691)	0.335
CAD	0.654 (0.233–1.833)	0.420	1.183 (0.534–2.618)	0.679
Dyslipidemia	1.005 (0.403–2.507)	0.992	1.155 (0.527–2.529)	0.719
CHF	1.244 (0.568–2.723)	0.585	0.896 (0.476–1.687)	0.734
MI	2.239 (0.881–5.687)	0.090	1.626 (0.804–3.287)	0.176
Ischemic stroke	1.578 (0.527–4.726)	0.415	1.264 (0.492–3.249)	0.627
ARU^b^	1.875 (0.841–4.184)	0.125	1.465 (0.767–2.799)	0.247
CADP-CT^b^	0.961 (0.408–2.267)	0.928	0.617 (0.310–1.226)	0.168
CEPI-CT^b^	0.779 (0.302–2.012)	0.606	1.421 (0.687–2.938)	0.343
GRS (rs3779647)^a^	1.110 (0.445–2.769)	0.822	0.909 (0.461–1.795)	0.784
DPP6 (rs1387180)^a^	0.979 (0.450–2.129)	0.958	0.716 (0.386–1.329)	0.290
RGS 7 (rs2502488)^a^	2.686 (0.992–7.270)	0.052	1.599 (0.788–3.244)	0.194
PLA2G7 (rs7756935)^a^	1.079 (0.482–2.415)	0.853	0.891 (0.453–1.750)	0.737
Chr 9p21.3 (rs10120688)^a^	0.773 (0.345–1.728)	0.530	0.703 (0.370–1.336)	0.282
TXBA2R (rs1131882)^a^	2.536 (1.149–5.597)	**0.021**	2.064 (1.056–4.037)	**0.034**
ADRA2A (rs4311994)^a^	0.209 (0.043–1.010)	0.052	0.799 (0.327–1.952)	0.622

Bold values indicates statistically significant differences

*HR* hazard ratio, *CI* confidence interval, *CAD* coronary artery disease, *CHF* congestive heart failure, *MI* myocardial infarction, *CEPI*-*CT* collagen/epinephrine closure time by PFA-100 method, *CADP*-*CT* collagen/adenosine diphosphate closure time by PFA-100 method, *ARU* aspirin reaction units for VerifyNow, *GRS* glutathione reductase, *DPP6* dipeptidyl peptidase like 6, *RGS7* regulator of G-protein signaling 7, *PLA2G7* lipoprotein-associated phospholipase A2 (Lp-PLA2)/plasma platelet-activating factor acetylhydrolase (PAF-AH), *Chr 9p21.3* chromosome 9p21.3, *TXBA2R* thromboxane A2 receptor, *ADRA2A* alpha-2A-adrenergic receptor

Bold values indicates statistically significant differences

^a^Measured for different closure time cutoff points (CEPI-CT < 193 s, CADP-CT < 90 s, VerifyNow ARU ≥ 550)

^b^Carriers of one or more minor allele (dominant model)

**Table 5:** Multivariate analysis for primary and secondary endpoint

Variables	Primary endpoint		Secondary endpoint	
	HR (95% Cl)	*p* value	HR (95% Cl)	*p* value
Age	1.09 (1.04–1.15)	0.0009	1.07 (1.03–1.11)	0.0008
TXBA2R (rs1131882)	2.34 (1.09–5.00)	0.028	1.89 (1.00–3.57)	0.048

*HR* hazard ratio, *CI* confidence interval, *TBXA2R* thromboxane A2 receptor

In univariate analysis, significant association was found in the dominant model of TXBA2R SNP (rs1131882) and age (Table 4). In univariate analysis, no connection was found between platelet reactivity measured with CADP-CT (*p* = 0.168), CEPI-CT (*p* = 0.343) and ARU (*p* = 0.247) and secondary endpoint (Table 4). Also in multivariate regression the dominant model of TXBA2R polymorphism (rs1131882) (*p* = 0.048) and age (*p* = 0.0008) was associated with presence of cardiovascular events (Table 5). Kaplan–Meier curve for the dominant model of rs1131882 polymorphism is shown in Fig. 1.

## Discussion

Our observational study identified potential association of a genomic variant, which was earlier described to be connected with platelet reactivity, and long-term prognosis in T2DM patients taking ASA. We found for the first time that genetic polymorphism within TXBA2R gene could be also linked to all-cause mortality in this high-risk population. In our study group of T2DM patients, the most significant results were obtained for rs1131882 located in the exonic part of TXBA2R gene in the short arm of chromosome 19 at position 13.3. Observed finding indicates that this particular polymorphism may be associated with increased risk of mortality, as magnitude of the observed effect for the TXBA2R genetic variant was significantly larger than for other genetic variants included in the study.

TXBA2R belongs to the superfamily of receptors for the eicosanoid thromboxane A2 (TxA2) and the isoprostane 8-iso-prostaglandin F2α [13]. Actions of thromboxane A2, a potent platelet activator and vasoconstrictor, are mediated by the activation of TXBA2R in the cell membrane; therefore, its role is crucial in platelet-involved processes [15]. Even though the rs11318632 SNP is synonymous and unlikely to influence the characteristics of the receptor directly, there are many potential mechanisms that may explain the biological influence of synonymous changes and its impact on multiple steps of gene expression, including its influence on mRNA processing, translation speed and transcription of unstable, easily degraded mRNA [16, 17].

The relationship between the TBXA2R polymorphism and platelet aggregation was previously analyzed in healthy volunteers [18–20]. Moreover, the association of the TBXA2R polymorphism (rs1131882) with residual platelet reactivity was also found in diabetic patients on ASA therapy, and these results were consistent with data from healthy volunteers described earlier by Postula et al. [2] and Fontana et al. [21]. It should be noted that some other TXBA2R genetic variants could have an impact on platelet’s function due to its decreased expression or modified function [14, 22]. For example, Nisar et al. [22] identified recently the new and functional genetic variant of TXA2 receptor, which was found to predict an asparagine to serine substitution (N42S) and cause platelet dysfunction due to reduced surface expression. Also Mumford et al. [14] demonstrated that another TBXA2R genetic variant, the D304N substitution, leads to clinically significant platelet dysfunction by reducing ligand binding.

Thus far, no study evaluated the potential association between platelet genetics and mortality in diabetic patients. The influence of platelet genetic profile on development of cardiovascular diseases in this population has been, however, reported previously. Wang et al. [23] analyzed three platelet SNPs (ITGA2-rs1062535, GNB3-rs5443 and MTHFR-rs1801133) in a large cohort of diabetic Chinese patients, but revealed no relationship between these polymorphisms and CAD or heart failure. On the other hand, the impact of ITGB3 PLA1A2 on survival in prediabetic patients was reported [24]. Several studies had also analyzed the influence of platelet genetics on survival or cardiovascular events in non-diabetic patients [25, 26]. Palmerini et al. revealed high on-treatment platelet reactivity in clopidogrel-treated patients with non-ST-segment elevation acute coronary syndrome (non-STEMI) undergoing percutaneous coronary intervention (PCI) had an influence on cardiac mortality, myocardial infarction and stent thrombosis in patients with high score in questionnaire assessing Synergy Between Percutaneous Coronary Intervention With Taxus And Cardiac Surgery. However, no association between ITGB3, 196 T>C polymorphism and these events were reported [25]. Also Zhang et al. [26] analyzed 5 platelets genes SNPs in ST-segment elevation acute coronary syndrome (STEMI) patients after PCI and showed that the F2R rs168753 minor allele was an independent predictor of the composite ischemic events after adjustment of established risk factors. It was also reported that the P2RY12 rs6809699 minor allele was an independent predictor of major bleedings.

Since diabetes mellitus is a common and complex multifactorial disorder, a large number of variants in the candidate genes may be involved, and every gene might only have a small to modest effect [2, 9]. Even though various genes known to be involved in platelet activation have been analyzed in the context of cardiovascular events, no study investigated the influence of TXBA2R gene on mortality specifically in diabetic population. It was previously established that hyperglycemia in platelets leads to calcium accumulation and increased release of proaggregant factors, which could explain why platelets from diabetic patients show accelerated response and increased aggregation compared with those from healthy subjects [27, 28]. Furthermore, both platelet reactivity and excretion of thromboxane metabolites are increased in obese patients with insulin resistance [29]. Faster recovery of cyclooxygenase activity despite ASA treatment was also reported in this group of subjects [30]. In addition, platelet hyperactivity can be explained by reduced sensitivity to agents exerting an inhibitory modulation of platelet responses, altered intracellular milieu with elevated cytosolic Ca2+, intensified thromboxane A2 synthesis and an increased number and function of GPIIb/IIIa complexes on platelet membranes [31]. In our previous studies, we also found that in some diabetic patients reactivity can be increased despite ASA therapy and modulated by both clinical and biochemical variables [2, 9, 32, 33]. However, in the current investigation we failed to find any association between platelet function and long-term cardiovascular events prognosis [34]. One of the potential explanations is that in the analysis we included only baseline measurement of platelet reactivity. It has been reported that platelet hyperactivity could change not only during a day but also in long-term observation; hence, our results may not represent possible changes of platelet reactivity over time related to biochemical and metabolic disturbances [2, 9, 32, 33, 35–40].

In our study, platelet reactivity was found to have any influence on neither primary nor secondary endpoint. However, this association was reported in several previous studies. Chiu et al. [41] observed that platelet reactivity after ASA or clopidogrel treatment (CEPI-CT < 193 s and CADP-CT < 95 s) was independently predictive of cardiovascular death, stroke and myocardial infarction after 24 months in patients undergoing percutaneous coronary intervention. Also a large meta-analysis performed in a similar group of 3059 patients revealed that high on-treatment platelet reactivity around the time of PCI is associated with long-term cardiovascular events including death, MI and stent thrombosis [42]. The differences between results of our study and results of the meta-analysis may stem from several factors. Firstly, the data combined in the meta-analysis were based on patients who had suffered from acute cardiovascular events, whereas the subjects of our study were affected by chronic disease. Secondly, different techniques were used to assess platelet reactivity in our investigation and in reports included in the meta-analysis. These factors can significantly contribute to differences in results and should be taken into consideration.

The major limitation of our study is a relatively modest significance of the observed effect of the candidate locus for this study does not exclude the risk of false positive results due to multiple testing, well below the significant threshold commonly accepted for statistical significance in GWAS, i.e., 5 × 10–8 which is related to the relatively small effect size and small size of the study cohort, as well as relatively high number of patients lost in follow-up. The method of collecting and scrutinizing the follow-up may be to blame for this limitation. Due to long observational period, personal contact and examination of the patient was difficult. Since it is an observational study that was performed on a population with specific study entry criteria, a small number of events were included in primary or secondary endpoint and not all clinical data or causes of mortality could be incorporated into the analysis. Because insufficient information is included in the Polish Statistical Registries, we were unable to obtain explicit details on the cause of death of the subject and defined primary end point as all-cause mortality instead of cardiovascular death. Another important limitation is related to the fact that only some SNPs previously postulated to be associated with platelets activity in this population were included into the analysis. It is therefore possible that other might also alter primary or secondary endpoint. In our study, platelet reactivity was assessed with the use of three point-of-care tests (CEPI-CT and CADP-CT by PFA-100 and ARU by VerifyNow Aspirin Assay) employed in multiple centers, and not light transmission aggregometry (LTA), which is considered the gold standard for platelet reactivity evaluation [43]. Finally, only single results of platelet’s function tests were included into the analysis and hence, we were unable to analyze variations in platelet reactivity in long-term follow-up and their association with prognosis. Our current findings should be interpreted with cautiousness as they await further confirmation from more detailed studies.

We believe that understanding of the genetic background of platelet function and its impact on clinical outcomes could have a profound clinical ramification for personalizing platelet-directed pharmacotherapy, by providing insight into the risks and possible benefits associated with specific genotypes. Further studies in larger diabetic populations are necessary to confirm and extend the results we observed in this cohort.
